# Improved Dual Attention for Anchor-Free Object Detection

**DOI:** 10.3390/s22134971

**Published:** 2022-06-30

**Authors:** Ye Xiang, Boxuan Zhao, Kuan Zhao, Lifang Wu, Xiangdong Wang

**Affiliations:** 1 Faculty of Information Technology, Beijing University of Technology, Beijing 100124, China; xiangye@bjut.edu.cn (Y.X.); zhaoboxuan@emails.bjut.edu.cn (B.Z.); s201861077@emails.bjut.edu.cn (K.Z.); lfwu@bjut.edu.cn (L.W.); 2 School of Physical Education, Jimei University, Xiamen 361021, China

**Keywords:** anchor-free object detection, dual attention, adaptive weight assignment

## Abstract

In anchor-free object detection, the center regions of bounding boxes are often highly weighted to enhance detection quality. However, the central area may become less significant in some situations. In this paper, we propose a novel dual attention-based approach for the adaptive weight assignment within bounding boxes. The proposed improved dual attention mechanism allows us to thoroughly untie spatial and channel attention and resolve the confusion issue, thus it becomes easier to obtain the proper attention weights. Specifically, we build an end-to-end network consisting of backbone, feature pyramid, adaptive weight assignment based on dual attention, regression, and classification. In the adaptive weight assignment module based on dual attention, a parallel framework with the depthwise convolution for spatial attention and the 1D convolution for channel attention is applied. The depthwise convolution, instead of standard convolution, helps prevent the interference between spatial and channel attention. The 1D convolution, instead of fully connected layer, is experimentally proved to be both efficient and effective. With the adaptive and proper attention, the correctness of object detection can be further improved. On public MS-COCO dataset, our approach obtains an average precision of 52.7%, achieving a great increment compared with other anchor-free object detectors.

## 1. Introduction

Object detection algorithms have been considerably improved based on convolutional neural networks (CNNs). As a fundamental research in the field of computer vision, object detection can be utilized in various visual tasks, such as facial detection [1], object tracking [2], and so on. Currently there are still some challenges remaining in object detection, thus attracting much attention from researchers.

Deep learning based methods have become the mainstream in object detection [3,4], and existing correlative algorithms can be divided into two aspects: anchor-based detectors and anchor-free detectors. The anchor-based detectors [5,6,7] have developed for a long time and obtained great performance. Anchors, a set of predefined boxes with specific sizes and aspect ratios, are essential in anchor-based detectors, such as Faster R-CNN [8], SSD [9], and YOLOv4 [7]. The anchors are to be regressed to correct areas with the help of labels during training, which will reduce the difficulty of bounding boxes prediction. However, the hyper-parameters, e.g., sizes, aspect ratios, and the number of anchors, may have a great influence on the detection results and have to be designed carefully according to the dataset and the algorithm through adequate experiments.

**Figure 1:**
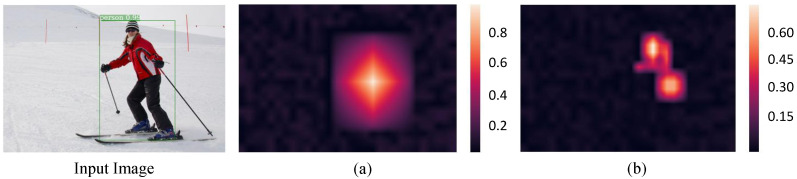
The visualization of weight assignment results for FCOS (**a**) and our algorithm (**b**).

Anchor-free detectors [10,11,12] do not involve the hyper-parameters setting related to anchors and aim to predict bounding boxes directly. In recent years, much attention has been drawn on anchor-free detectors, meanwhile a problem of low-quality detections often existing in results is also found. To tackle it, FCOS [13] and CornerNet [14] both propose to assign high weights to central regions and low weights to marginal regions within bounding boxes, as shown in Figure 1a. The low-quality detections that usually occur at locations far from object center can then be suppressed. Furthermore, a keypoint-based detector named CenterNet [15] is proposed. CenterNet predicts the center keypoint by gathering the max summed values along horizontal and vertical directions. It allows the center keypoints to perceive visual patterns within objects, and the correctness of bounding boxes can be identified consequently. These algorithms explore the weight assignment strategy and believe points near the center should be given more weights to produce high-quality detections. However, the central regions may include nothing of target objects or be shielded in some situations [16,17]. Hence we are to pursue an adaptive weight assignment strategy to obtain the more reasonable confidence scores automatically for predicted bounding boxes, as shown in Figure 1b.

The attention mechanism [18,19] offers a good option for learning weights adaptively, and has been widely utilized in various tasks such as image classification, detection and segmentation [20,21]. There are commonly two types of attention, i.e., the spatial attention and channel attention. Some algorithms [22] concentrate on improving either one of them to boost the performance, whereas more recent works [23,24,25] are inclined to explore the dual attention to model the richer interdependencies of features. To implement the dual attention mechanism and connect it with deep neural networks, two ways are often exploited: (i) estimate the channel attention and spatial attention successively [25] and (ii) estimate the dual attention in parallel and then fuse them [23,24]. For (i), channel attention is followed with spatial attention, which means channel attention will inevitably interfere spatial attention. Conversely, the parallel way is more likely to learn the effective spatial and channel attention weights directly from original deep features. For (ii), in spatial attention mechanism within a parallel framework, the standard convolutions are generally used, which actually involve interaction in both the spatial and channel dimensions. As a result, the spatial attention weights are confused with the channel attention weights.

In view of these deficiencies, we propose an improved dual attention mechanism and apply it for the adaptive weight assignment in anchor-free object detection. Firstly, we adopt the parallel way to implement the dual attention mechanism. In spatial attention, the depthwise convolution rather than standard convolution is used to untie the spatial and channel attention thoroughly, avoiding the confusion issue. In channel attention, the 1D convolutional layer performing local cross-channel interaction strategy, instead of fully connected layer, is used for higher computational efficiency and better performance. Through improved dual attention mechanism, proper confidence scores for object classification can be obtained adaptively. Since the confidence scores indicate the probability of being high-quality bounding boxes, the correctness of object detection can then be enhanced through NMS [26]. Experiments are conducted on the MS-COCO dataset [27], a challenging benchmark for large-scale object detection. It demonstrates that the proposed algorithm can achieve competitive performance, in contrast with other anchor-free object detectors.

The contributions of this paper are summarized as follows:

- We introduce the adaptive weight assignment based on dual attention mechanism into anchor-free object detector. It can obtain the adaptive distribution of classification confidence scores automatically.
- We improve the dual attention mechanism designed for other tasks and apply it to object detection. The improved dual attention mechanism can prevent the confusion between spatial and channel attention, and is both efficient and effective.
- The experimental results on public MS-COCO dataset demonstrate that the proposed algorithm can achieve the state-of-the-art performance comparing with other anchor-free object detectors.

## 2. Related Work

There are commonly two kinds of object detection algorithms: anchor-based detectors and anchor-free detectors. Many of them improve performance based on the attention.

Anchor-based detectors. The anchor boxes, which are placed densely on feature maps to adjust and refine prediction results for obtaining the final bounding boxes, are widely used in anchor-based detectors. Faster R-CNN [8] introduced the region proposal network (RPN) with anchors for regions of interest(RoIs) generation. Mask R-CNN [28] appended a mask generation branch to Faster R-CNN [8] for instance segmentation. Cascade R-CNN [29] learned a multi-stage detector by increasing the IoU threshold for more accurate prediction. YOLOv2, YOLOv3, and YOLOv4 [5,6,7] have been the representative real-time object detection algorithms which also involve the anchors. Another real-time detector SSD [9] assigned the dense anchors on feature maps for object detection with various sizes. RetinaNet [30], which involved a great number of anchors, adopted the focal loss for the problem of imbalance between positive and negative samples. At present, many anchor-based detectors show competitive performance.

Anchor-free detectors. Anchor-free detectors do not require the dense assignment of anchors. CornerNet [14] generated the bounding box through a pair of corners. Based on CornerNet, CenterNet [15] added a center prediction branch to filter the false boxes. CornerNet-Lite [31] combined CornerNet-Saccade and CornerNet-Squeeze to improve both the efficiency and accuracy. The four extreme points of objects were utilized and predicted in ExtremeNet [10]. Zhu et al. [12] predicted the center points and the sizes of bounding boxes for object detection. MatrixNets [32] provided a new deep architecture which can handle objects with different sizes and aspect ratios. ResPoints [11] regarded the object as a fixed number of points that were combined in specific manners for predicted box. YOLOv1 [33] divided the whole image with *S × S* grids and regressed the boxes through grid cells containing the centers of objects. DenseBox [34] and UnitBox [35] generated results with the distances from positive points to boundaries of boxes. For positive points, PPDet [17] provided a new definition to implement the relaxed labeling strategy aimed to reduce the label noise in anchor-free detectors. In FSAF [36], an anchor-free branch was attached to RetinaNet [30] for online feature level selection. FCOS [13] equipped with GroupNorm [37] and GIoU loss [38], innovatively introduced the centerness strategy, which assigned the central region with the highest weight to improve the quality of bounding boxes. DDBNet [16] dived deeper into box regressions of center key-points and takes care of semantic consistencies of center key-points.

Attention weight aware. The attention module [18,19] has been widely used in object detection. For the attention in channel dimension, SENet [39] employed the Squeeze and Excitation to learn the weights of channels. SKNet [40] exploited two branches with different kernel sizes to learn the channel weights. ECANet [22] improved the performance of SENet with fewer parameters, which replaced the fully-connected layers with 1D convolutional layers that have dynamic kernel sizes. ResNeSt [41] introduced the split-attention block with the combination of group convolution and channel attention mechanism. The attention modules in these algorithms can be extensively utilized in visual classification, detection, tracking, and segmentation tasks by combining with the backbone network, e.g., ResNet [42]. In this paper, we will design a dual attention module, and add it into object detection network to learn the spatial and channel weights for classification confidence scores.

## 3. The Proposed Approach

### 3.1. Overview

**Figure 2:**
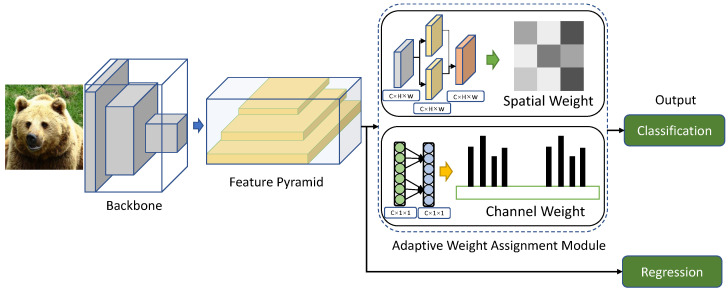
The framework of the proposed object detection algorithm. It mainly includes the backbone, feature pyramid, adaptive weight assignment based on dual attention, regression, and classification. The adaptive weight assignment module based on dual attention involves both spatial weight and channel weight.

We firstly introduce the whole framework of the proposed object detection approach, as shown in Figure 2. There are five components in total: backbone, feature pyramid, adaptive weight assignment based on dual attention, regression and classification. The backbone is to extract the deep features of objects, and commonly adopts the ResNet-50 [42] or ResNet-101 [42]. The feature pyramid network [43] is utilized to integrate the features of high levels and low levels. In both anchor-based detectors (e.g., RetinaNet [30] and Faster R-CNN [8]) and anchor-free detectors (e.g., FSAF [36] and FCOS [13]), it has been proved that the feature pyramid can enhance the detection performance by a large margin. We employ five levels {*P*${}_{3}$, *P*${}_{4}$, *P*${}_{5}$, *P*${}_{6}$, *P*${}_{7}$} in the feature pyramid with strides of {8, 16, 32, 64, 128}, separately. The adaptive weight assignment module based on dual attention aims at weighting confidence scores adaptively for bounding boxes, and is the major contribution of this paper. There are two parallel branches in this module, i.e., the spatial weight assignment and the channel weight assignment. Lastly, the regression subnet and classification subnet follow with feature pyramid and adaptive weight assignment, respectively. The regression subnet is used for size and position prediction of bounding boxes, and the classification subnet is used for confidence scores prediction of bounding boxes.

Regression subnet. The regression subnets are attached to every feature level to predict the corresponding bounding boxes. A regression subnet consists of four 3 × 3 convolutional layers and outputs the feature maps with four channels for regression. Take the *i*th level of feature pyramid as an example, we can generate the feature maps of shape (*4*, *H*${}_{i}$, *W*${}_{i}$) for regression. (*H*${}_{i}$, *W*${}_{i}$) is the size of feature maps, and four channels indicate the distances from one specific position to the four boundaries of a bounding box. Given a position and its corresponding four values, a candidate bounding box can then be obtained.

Classification subnet. The classification subnets are also attached to every feature level to estimate the classification scores. For the *i*th level of feature pyramid, we can generate the feature maps with shape (*C*, *H*${}_{i}$, *W*${}_{i}$) for classification. *C* denotes the channel number as well as the category number, which is 80 for the MS-COCO dataset [27]. On the feature maps for classification, if one position falls into a ground-truth bounding box, it can be regarded as the positive sample, otherwise it is the negative sample. Moreover, it is worth noting that at the high level of feature pyramid, some small bounding boxes may never appear in classification output. Accordingly, following FCOS [13], we assign different ground-truth bounding boxes to different levels of feature pyramid. Specifically, for the five levels {*P*${}_{3}$, *P*${}_{4}$, *P*${}_{5}$, *P*${}_{6}$, *P*${}_{7}$}, we define a set of threshold values {*m*${}_{2}$, *m*${}_{3}$, *m*${}_{4}$, *m*${}_{5}$, *m*${}_{6}$, *m*${}_{7}$} as {0, 64, 128, 256, 512, +*∞*}. If *m${}_{i-1}$* < max(*l*, *r*, *t*, *b*) < *m*${}_{i}$, where max(*l, r, t, b*) represents the maximum distance to the boundaries of a certain ground-truth bounding box, the certain ground-truth bounding box will be assigned to the *i*th feature level.

### 3.2. Adaptive Weight Assignment Based on Dual Attention

**Figure 3:**
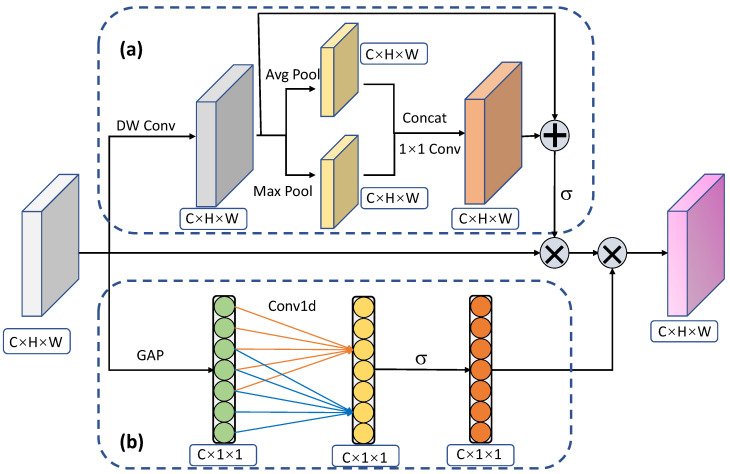
The structure of adaptive weight assignment module based on improved dual attention. It is mainly composed of two parts: (**a**) spatial weight attention and (**b**) channel weight attention. DW Conv means the depthwise convolution. Avg Pool and Max Pool mean average pooling and max pooling, respectively. GAP means global average pooling. σ means the sigmoid activation.

The structure of adaptive weight assignment module based on improved dual attention mechanism is shown in Figure 3. It consists of two components: (a) spatial weight attention and (b) channel weight attention. The adaptive weight assignment module is added right before the classification subnet so that the confidence scores can be adjusted with the adaptive weights.

Spatial weight attention. In Figure 3, the part of (a) represents the spatial weight attention. To obtain the spatial weights, existing algorithms usually employ the standard convolution. However, standard convolution involves the interaction in both spatial and channel dimensions, which will lead to the spatial weights being confused with the channel weights. Hence we propose to take the depthwise convolution in which each convolution kernel only operates on one channel of feature map. By separating spatial weight attention and channel weight attention thoroughly, the depthwise convolution can help learn a more reasonable distribution of confidence scores. Assume the input feature maps have the shape of (C,H,W), then the output of depthwise convolution denoted as $F_{a}^{1}$ also has the shape of (C,H,W). The calculation is formulated as:

(1) $$F_{a}^{1}\left(c\right)=V\left(c\right)*X\left(c\right),$$

where *X*(*c*), and *F${}_{a}^{1}$*(*c*) indicate the *c*th channel for input and output feature maps, respectively, and *V*(*c*) is a 2D spatial kernel for the *c*th channel.

After depthwise convolution, pooling operation is utilized to further extract salient features. We adopt two types of pooling operations, i.e., the average pooling and max pooling, to process the *F${}_{a}^{1}$*, respectively. The output feature maps of average pooling and max pooling have the same shapes, and are concatenated together to obtain the new feature maps *F${}_{a}^{2}$* as follows:

(2) $$F_{a}^{2}=Concat\left(MaxPool\left(F_{a}^{1}\right),AvgPool\left(F_{a}^{1}\right)\right),$$

where *MaxPool* and *AvgPool* refer to the max pooling and average pooling, and *F${}_{a}^{2}$* has the shape of (*2C, H, W*). Afterwards, the *F${}_{a}^{2}$* is transformed to *F${}_{a}^{3}$* with the shape of (*C, H, W*) through a *1×1* convolutional layer.

The operation of pooling especially the max-pooling usually loses some valuable information of feature maps, e.g., the abundant spatial relationship between local regions. To compensate for the lost information, the residual structure is needed. Hence we add the *F${}_{a}^{1}$* and *F${}_{a}^{3}$* to generate the new feature maps *F${}_{a}^{4}$*. The *F${}_{a}^{4}$* is lastly processed by a sigmoid function to make the value of each position on feature maps limited between 0 and 1. With the feature maps of spatial weights, we just multiply it by the *F${}_{a}^{1}$* to obtain the output *F${}_{a}^{5}$*.

Channel weight attention. Since different information may exist across different channels, the interaction in channel dimension is also vital for attention estimation. As shown in Figure 3, the part of (b) indicates the channel weight attention, which aims to calculate the reasonable weights distribution across channels. Specifically, the GAP means the global average pooling. Following SENet [39] and ECANet [22], to learn the weight information about channels, the global average pooling needs to be firstly performed as follows:

(3) $$F_{b}^{1}=g\left(X\right)=\frac{1}{H\times W}\sum_{i=1}^{H}\sum_{j=1}^{W}X_{ij},$$

where *H* and *W* represent the height and width of input feature maps *X*, and *F${}_{b}^{1}$* has the shape of (*C, 1, 1*).

Next, the Conv1d which means the 1D convolutional layer is utilized to learn the relations between different channels. Recently, the group convolution has been extensively used in various classification and detection networks with great performance gain. Hence, following [22], we opt to take the 1D convolutional layer instead of fully connected layer for better performance and fewer parameters. Concretely, the *F${}_{b}^{1}$* is fed into a 1D convolutional layer with a fixed kernel size of 5. The output is further processed by a sigmoid activation function to constrain the values to be between 0 and 1, obtaining *F${}_{b}^{2}$*. The formula can be written as:

(4) $$F_{b}^{2}=\sigma \left(Conv1d\left(F_{b}^{1}\right)\right)=\sigma \left(Conv1d\left(g\left(X\right)\right)\right),$$

where σ refers to the sigmoid activation function. At last, the *F${}_{a}^{5}$* for spatial weight attention is multiplied by the *F${}_{b}^{2}$* for channel weight attention, obtaining the final feature maps with shape of (*C, H, W*) for the adaptive weight assignment module.

**Figure 4:**
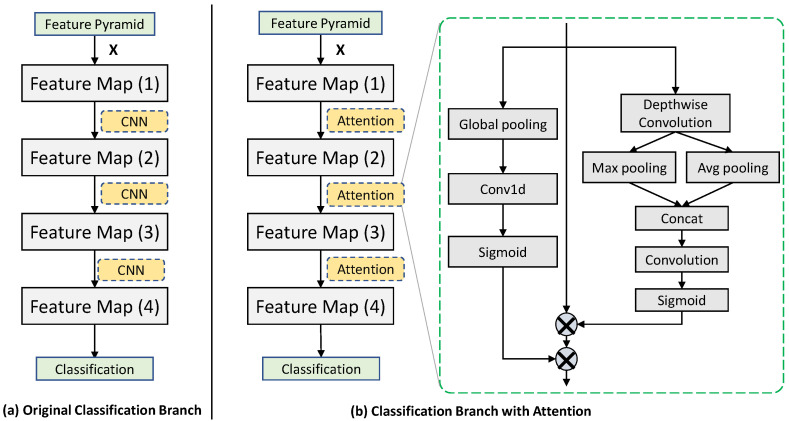
The comparison of (**a**) original classification branch and (**b**) modified classification branch with stacked dual attention. In (**a**), the standard convolutional layers are used. In (**b**), three standard convolutional layers are replaced by the three stacked dual attention modules.

Stacked dual attention. We stack the multiple modules of dual attention to further improve the performance. As shown in Figure 4a, the classification branch commonly used in existing algorithms [44] is a fully convolutional network, which consists of four standard convolutional layers with the last one for score calculation. We replace the first three standard 3 × 3 convolutional layers with the three stacked dual attention modules, constituting a new classification branch, as shown in Figure 4b. For fair comparison, the same layers of original classification branch and modified classification branch have the same size of output feature maps.

### 3.3. Loss

Our overall loss for training is

(5) $$L=\lambda_{1}L_{reg}+\lambda_{2}L_{cls},$$

where $L_{reg}$ and $L_{cls}$ denote the regression loss and classification loss separately, $\lambda_{1}$ and $\lambda_{2}$ are the corresponding weights and are both set as 1 in our experiments.

We take the IoU loss for regression. The $L_{reg}$ can then be written as

(6) $$L_{reg}=\frac{1}{N}\sum_{i=1}^{H}\sum_{j=1}^{W}-log\left(IoU\left(P_{ij},Y_{ij}\right)\right),$$

where *N* indicates the number of positive samples, *P${}_{ij}$* and *Y${}_{ij}$* are predicted bounding box and ground-truth bounding box on position (*i*, *j*), respectively, and *IoU* means the operation of intersection over union. Note that only the positive samples are counted. The regression loss for negative samples is immediately set to 0.

We use the focal loss [30] for classification. The $L_{cls}$ can be written as

$$L_{cls}=\frac{1}{N}\sum_{i=1}^{H}\sum_{j=1}^{W}\sum_{c=1}^{C}\left\{\begin{array}{cc} -\theta {\left(1-P_{ijc}\right)}^{\eta }log\left(P_{ijc}\right), & \mathrm{if}\;Y{}_{ijc}=1\\ -\left(1-\theta \right)P_{ijc}^{\eta }log\left(1-P_{ijc}\right), & \mathrm{otherwise} \end{array}\right.$$

where *N* is the total number of samples including positive and negative samples, *P${}_{ijc}$* and *Y${}_{ijc}$* are predicted score and ground-truth score, respectively, and θ and η are hyper-parameters which are set to 0.25 and 2 separately. The focal loss concentrates more on the easily misclassified samples, thus can alleviate the samples imbalance issue and gain better performance.

## 4. Experiment

The proposed algorithm in this paper is evaluated on the MS-COCO dataset [27] which includes over 1.5 million images with 80 categories. Section 4.1 gives training and testing details. Section 4.2 introduces visualized analysis for our proposed algorithm adopting adaptive weight assignment strategy. Section 4.3 presents the results of experiments.

### 4.1. Implementation Details

Training details: The backbone networks utilized in the proposed algorithm for training include ResNet-50 and ResNet-101 [42], which are pretrained on the ImageNet dataset. The maximum number of iterations is set as 200 K, and the initial learning rate is set to 5 × 10${}^{-3}$. The learning rate will be divided by 10 each time up to the 120 K and 160 K iterations. As for the input image, we resize it to 800 on the shorter side and less than or equal to 1333 on the longer side. The proposed algorithm is trained on 2 TitanX GPUs with a batch size of 8. On the MS-COCO [27] dataset, the split called trainval35K is used for training, which contains 80 K images for training set and 35 K images for validation set.

Testing details: After obtaining the predicted bounding boxes with specific categories through regression and classification subnets, we utilize the technique of non-maximum suppression (NMS) [45] to filter out the redundant predicted bounding boxes with high Intersection over Union (IoU) but low confidence score. The experimental results are evaluated through six metrics, namely AP, AP${}_{50}$, AP${}_{75}$, AP${}_{S}$, AP${}_{M}$ and AP${}_{L}$. AP is averaged over multiple IoU values and the IoU thresholds are from 0.5 to 0.95, which acts as the main evaluation indicator. AP${}_{50}$ and AP${}_{75}$ refer to IoU = 0.5 and 0.75, respectively. It is obvious that the larger the IoU threshold, the stricter the corresponding evaluation metric. AP${}_{S}$, AP${}_{M}$ and AP${}_{L}$ represent the average precision over different sizes of objects: area < 32${}^{2}$, 32${}^{2}$ < area < 96${}^{2}$ and area > 96${}^{2}$.

### 4.2. Visualized Analysis

As mentioned in Section 3, the proposed improved dual attention mechanism is added before the classification subnet to obtain adaptive weight distribution. In this section, we take an example to visualize the generated confidence scores for classification with different weight distributions and further analyze the impact on object detection.

**Figure 5:**
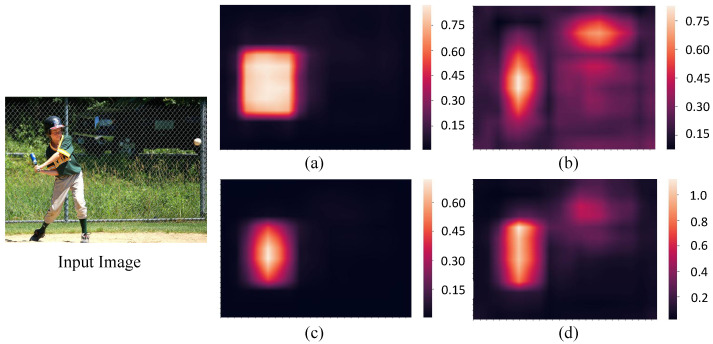
The visualization of classification confidence scores with different weight distributions. The left shows input image. (**a**) Shows the original classification confidence scores without weight assignment. (**b**) Shows the weight distribution of centerness. (**c**) Shows the classification confidence scores with weight distribution of centerness. (**d**) Shows the classification confidence scores with our proposed attention mechanism.

As shown in Figure 5, an athlete in input image is to be detected. Figure 5a shows the confidence scores for classification without any weight assignment. Figure 5b illustrates the weight distribution strategy named centerness [13], which assigns the center area of bounding box with high weight and the marginal area with low weight. Figure 5c shows the classification confidence scores with centerness strategy, which is obtained through multiplying the original scores by weights from centerness. Figure 5d shows the classification confidence scores with our proposed attention mechanism.

It can be seen that in Figure 5a, almost the whole region of athlete gains similar high scores, including the marginal area. With the high scores, some low-quality bounding boxes predicted at positions far away from the center of an object will also be preserved, leading to the detection performance degradation. In Figure 5c that shows the classification scores with centerness, the positions far away from central area tend to have lower confidence scores, which will restrain the low-quality bounding boxes to some extent. However, the central area is not as distinguishable as areas such as the head for athlete in input image, and does not deserve the highest score. In Figure 5d that shows the classification scores with our proposed attention mechanism, the area with highest score has an adaptive central deviation, and focuses on the athlete head that is the most recognizable part. Hence the results of Figure 5 demonstrate that our proposed dual attention mechanism does implements the adaptive weight assignment strategy, and can help improve the quality of predicted bounding boxes.

### 4.3. Experimental Results

In this section, the experimental results for verifying effectiveness of improved dual attention, ablation study and comparison with the state-of-the-arts are displayed and analyzed.

#### 4.3.1. Effectiveness of Improved Dual Attention

Different from the original dual attention mechanism, our proposed improved version adopts the depthwise convolution instead of standard convolution for spatial weight attention, and the 1D convolution instead of fully connected layer for channel weight attention. The effectiveness of the two modifications are verified separately below.

Firstly, for spatial weight attention, we take ResNet-50 and ResNet-101 as the backbone network in turn, and compare the standard convolution with the depthwise convolution under serial and parallel dual attention frameworks, respectively. The results are shown in Table 1. It can be clearly seen that the parallel framework is slightly better than the serial framework, and the depthwise convolution greatly improves the standard convolution, whether using ResNet-50 or ResNet-101. In our analysis, the depthwise convolution brings more performance gains than the parallel framework, which may be because the former can distinguish the spatial and channel attentions more thoroughly, and solve the issue of mutual interference more effectively.

Secondly, for channel weight attention, we compare the fully connected layer and the 1D convolution by using ResNet-50 as the backbone network and adopting the parallel framework. The results of AP and Frames-per-Second (FPS) are listed in Table 2. It shows that the 1D convolution achieves a significant performance improvement over the fully connected layer, reaching 4.4%. Such disparity in performance may be because the fully connected layer contains many more parameters and tends to suffer the overfitting problem. Meanwhile, the inference speed of 1D convolution is much faster than fully connected layer. Hence the 1D convolution is adopted for both higher efficiency and better performance.

#### 4.3.2. Ablation Study

Loss weights: Our overall loss for training is composed of regression loss and classification loss. To determine the appropriate weights, we experiment with different $\lambda_{1}$ and $\lambda_{2}$. The results of proposed algorithm taking ResNet-50 as backbone on the MS-COCO benchmark are shown in Table 3. It can be seen that the best performance is achieved by $\lambda_{1}$ = 1.0 and $\lambda_{2}$ = 1.0. Imbalanced loss weights cause the significant performance degradation. Hence we set $\lambda_{1}$ and $\lambda_{2}$ both to 1.

Framework design: There are two frameworks, i.e., the serial and parallel frameworks, that can be used for the proposed improved dual attention mechanism. We adopt them, respectively, for comparison. The experimental results are shown in Table 4. It demonstrates that both spatial weight attention and channel weight attention contribute a lot to the performance improvement, whether using serial or parallel framework. In addition, the channel weight attention plays a good complementary role to the spatial weight attention and effectively improves the performance (5.6% under serial framework and 5.2% under parallel framework), which indicates that information exchange between the channels is also significant for representation learning.

#### 4.3.3. Comparison with the State of the Art

Our proposed algorithm is evaluated on the MS-COCO benchmark to make comparison with the state-of-the-art anchor-based and anchor-free object detection algorithms, as shown in Table 5. It can be seen that our anchor-free algorithm achieves the results of 47.1% and 52.7% with the backbone of ResNet-50 and ResNet-101, respectively. Ours with backbone of ResNet-101 even outperforms most of the anchor-based detectors, and is competitive with YOLOv4-P7 [46] (55.5%) and EfficientDet-D7 [47] (53.7%). Both the two algorithms use the particular backbones for their own network structures, while ours adopts the common backbone, i.e., ResNet, and will be probably improved as the backbone network is updated. For anchor-free object detection, ours with backbone of ResNet-101 defeats all the other algorithms, and achieves the substantial improvements. For example, ours is better than the best keypoint-based algorithm RepPoints [11] with a 7.7% increase in accuracy (52.7% vs. 45.0%). The comparison results with state-of-the-art detectors fully demonstrate the effectiveness of our proposed algorithm with adaptive weight assignment strategy and modified parallel spatial and channel weight attention framework.

## 5. Conclusions

In this paper, we have proposed an adaptive weight assignment strategy based on improved dual attention mechanism for anchor-free object detection. It can help learn a more reasonable distribution of weights and reduce the low-quality detections by untying the dual attention effectively and efficiently, in case that the central area includes nothing of target object or is just shielded. For implementation of the dual attention mechanism, we develop a new parallel framework with the depthwise convolution for spatial attention and the 1D convolution for channel attention. The depthwise convolution can untie the spatial and channel attention thoroughly and help avoid the confusion issue. The 1D convolution can enhance the performance as well as the computational efficiency. On the MS-COCO dataset, our proposed algorithm is shown to achieve a great increment compared with other anchor-free object detectors.

## Figures and Tables

**Table 1:** Comparison between standard convolution and depthwise convolution under serial and parallel dual attention frameworks. The backbones we used are ResNet-50 and ResNet-101.

Method	Framework	Backbone	AP
standard convolution	Serial	ResNet-50	45.0
depthwise convolution	Serial	ResNet-50	46.7
standard convolution	Parallel	ResNet-50	45.2
depthwise convolution	Parallel	ResNet-50	47.1
standard convolution	Serial	ResNet-101	49.0
depthwise convolution	Serial	ResNet-101	52.2
standard convolution	Parallel	ResNet-101	49.3
depthwise convolution	Parallel	ResNet-101	52.7

**Table 2:** Comparison between fully connected layer and 1D convolution for channel weight attention.

Method	Backbone	AP	FPS
fully connected layer	ResNet-50	42.7	9.8
1D convolution	ResNet-50	47.1	13.3

**Table 3:** Comparison between weights for regression loss and classification loss.

$\lambda_{1}$	$\lambda_{2}$	AP
1.0	1.0	47.1
1.0	2.0	43.6
2.0	2.0	43.5

**Table 4:** Comparison between different frameworks for proposed improved dual attention module.

Channel Weight	Spatial Weight	Framework	AP
		Serial	33.1
	✓	Serial	41.3
✓	✓	Serial	46.9
		Parallel	33.5
	✓	Parallel	41.9
✓	✓	Parallel	47.1

**Table 5:** Comparison with the state-of-the-art detectors on MS-COCO benchmark. The backbones include ResNet (R) [42], DetNet (D) [48], ResNeXt (X) [49], Dual Path Network (DPN) [50], Cross Stage Partial (CSP) [51], EfficientNet (E) [52], and Hourglass (H) [53].

Method	Backbone	AP	AP${}_{50}$	AP${}_{75}$	AP${}_{S}$	AP${}_{M}$	AP${}_{L}$
Anchor-based detectors:							
Faster R-CNN [8]	R-101	36.2	59.1	39.0	18.2	39.0	48.2
DetNet [48]	D-59	40.3	62.1	43.8	23.6	42.6	50.0
Soft-NMS [54]	R-101	40.8	62.4	44.9	23.0	43.4	53.2
C-Mask R-CNN [55]	R-101	42.0	62.9	46.4	23.4	44.7	53.8
Cascade R-CNN [29]	R-101	42.8	62.1	46.3	23.7	45.5	55.2
Libra R-CNN [56]	X-101-64×4d	43.0	64.0	47.0	25.3	45.6	54.6
Revisting R-CNN [57]	R-101+R-152	43.1	66.1	47.3	25.8	45.9	55.3
SNIP [58]	DPN-98	45.7	67.3	51.1	29.3	48.8	57.1
TridentNet [59]	R-101-DCN	46.8	67.6	51.5	28.0	51.2	60.5
RefineDet512 [60]	R-101	36.4	57.5	39.5	16.6	39.9	51.4
RetinaNet [30]	R-101	39.1	59.1	42.3	21.8	42.7	50.2
YOLOv4 [7]	CSPDarkNet-53	43.5	65.7	47.3	26.7	46.7	53.3
YOLOv4-P7 [46]	CSP-P7	55.5	73.4	60.8	38.4	59.4	67.7
EfficientDet-D7 [47]	E-B6	53.7	72.4	58.4	35.8	57.0	66.3
Anchor-free detectors:							
ExtremeNet [10]	H-104	40.2	55.5	43.2	20.4	43.2	53.1
CornerNet [14]	H-104	40.5	56.5	43.1	19.4	42.7	53.9
CenterNet-HG [12]	H-104	42.1	61.1	45.9	24.1	45.5	52.8
Grid R-CNN [61]	X-101	43.2	63.0	46.6	25.1	46.5	55.2
CornerNet-Lite [31]	H-54	43.2	-	-	24.4	44.6	57.3
CenterNet [15]	H-104	44.9	62.4	48.1	25.6	47.4	57.4
RepPoints [11]	R-101-DCN	45.0	66.1	49.0	26.6	48.6	57.5
FoveaBox [62]	X-101	42.1	61.9	45.2	24.9	46.8	55.6
FSAF [36]	X-101-64×4d	42.9	63.8	46.3	26.6	46.2	52.7
FCOS [13] w/imprv	X-101-64×4d	44.7	64.1	48.4	27.6	47.5	55.6
SAPD [63]	R-101-DCN	46.0	65.9	49.6	26.3	49.2	59.6
ATSS [64]	X-101-DCN	47.7	66.5	51.9	29.7	50.8	59.7
Ours	R-50	47.1	65.7	51.1	28.2	50.0	58.7
Ours	R-101	52.7	71.1	56.9	35.0	56.0	66.1

## Data Availability

The dataset analyzed during the current study is available in the MS-COCO repository [27] with the identifier accessed on 1 August 2021 “https://cocodataset.org”, which can be used with permission of COCO Consortium.
